# Periadventitial Application of Rapamycin-Loaded Nanoparticles Produces Sustained Inhibition of Vascular Restenosis

**DOI:** 10.1371/journal.pone.0089227

**Published:** 2014-02-21

**Authors:** Xudong Shi, Guojun Chen, Lian-Wang Guo, Yi Si, Men Zhu, Srikanth Pilla, Bo Liu, Shaoqin Gong, K. Craig Kent

**Affiliations:** 1 Department of Surgery, University of Wisconsin Hospital and Clinics, Madison, Wisconsin, United States of America; 2 Wisconsin Institutes for Discovery, University of Wisconsin, Madison, Wisconsin, United States of America; 3 Materials Science Program, University of Wisconsin, Madison, Wisconsin, United States of America; 4 Department of Biomedical Engineering, University of Wisconsin, Madison, Wisconsin, United States of America; The Ohio State University, United States of America

## Abstract

Open vascular reconstructions frequently fail due to the development of recurrent disease or intimal hyperplasia (IH). This paper reports a novel drug delivery method using a rapamycin-loaded poly(lactide-co-glycolide) (PLGA) nanoparticles (NPs)/pluronic gel system that can be applied periadventitially around the carotid artery immediately following the open surgery. *In vitro* studies revealed that rapamycin dispersed in pluronic gel was rapidly released over 3 days whereas release of rapamycin from rapamycin-loaded PLGA NPs embedded in pluronic gel was more gradual over 4 weeks. In cultured rat vascular smooth muscle cells (SMCs), rapamycin-loaded NPs produced durable (14 days versus 3 days for free rapamycin) inhibition of phosphorylation of S6 kinase (S6K1), a downstream target in the mTOR pathway. In a rat balloon injury model, periadventitial delivery of rapamycin-loaded NPs produced inhibition of phospho-S6K1 14 days after balloon injury. Immunostaining revealed that rapamycin-loaded NPs reduced SMC proliferation at both 14 and 28 days whereas rapamycin alone suppressed proliferation at day 14 only. Moreover, rapamycin-loaded NPs sustainably suppressed IH for at least 28 days following treatment, whereas rapamycin alone produced suppression on day 14 with rebound of IH by day 28. Since rapamycin, PLGA, and pluronic gel have all been approved by the FDA for other human therapies, this drug delivery method could potentially be translated into human use quickly to prevent failure of open vascular reconstructions.

## Introduction

Over a million vascular reconstructions including more than 300,000 conventional open surgical interventions are performed in the USA each year to treat cardiovascular disease. Unfortunately a large number of these eventually fail due to the development of restenosis or intimal hyperplasia (IH). Despite our in depth understanding of this process and the development of inhibitors, treatments have lagged behind because of the lack of an effective method of drug delivery; this is particularly true for open vascular surgery where there are currently no clinically available methods to prevent recurrent vascular disease. Although systemic drug delivery has been attempted, toxicity has limited its success [1]. In addition systematic therapy cannot provide sufficient therapeutic drug levels at the target artery for a long time. To maintain effective drug concentrations without toxicity, local application is the optimal approach.

Advances in local drug delivery have been made for percutaneous vascular interventions. Both paclitaxel and rapamycin have advanced to clinical use and are currently applied *via* stents or balloons following percutaneous angioplasty. Although this approach has limitations including an increased risk of thrombosis, with the use of these stents, the rate of restenosis has diminished by at least 50% [2]. However, drug-eluting stents are not applicable in the case of open surgical procedures such as bypass, endarterectomy or dialysis access. For these procedures there currently are no viable clinical options for the prevention of restenosis. The result is an unmet clinical need for an effective method of drug delivery following open surgical revascularization. The absence of a technique for drug delivery following open surgery is surprising since the challenges of remote drug delivery following percutaneous angioplasty would seem more formidable than those for open surgery. At the time of open vascular reconstruction, the treated vessel is readily accessible making application of drug more direct and easily achievable. Periadventitial drug delivery has additional advantages, including minimized effect of the drugs on luminal endothelial cell growth due to the creation of a gradient resulting in diminished luminal drug concentrations.

Rapid progress in the field of nanomedicine in recent years offers new promising approaches to diagnose and treat many major diseases including cancers, vascular diseases, infections (*e.g*., HIV, malaria, tuberculosis), metabolic disease (*e.g.*, diabetes and osteoporosis), and autoimmune diseases (*e.g*., glaucoma) [3]–[5]. Nanoparticles (NPs) encompass a variety of submicron colloidal nanosystems that may be inorganic, liposome-based, or polymer-based. Poly(lactic-co-glycolic acid) (PLGA) NPs are likely the most widely studied drug delivery NPs for the treatment of a broad range of diseases [4], [6], [7]. The popularity of PLGA NPs can be attributed to a number of factors. (1) PLGA has excellent biocompatibility and has been approved by FDA. (2) The biodegradability of PLGA and similarly, the drug release profiles of PLGA NPs can be conveniently engineered from weeks to months by controlling the chemical structure of PLGA (LA/GA ratio), its molecular weight, and the size of the NPs *etc* [8]. PLGA NPs can be used to encapsulate either hydrophobic drugs or hydrophilic drugs (*e.g*., nucleic acids and proteins) using well-established emulsion processes. (4) PLGA NPs can provide local, site-specific, and sustained and controlled drug release. (5) PLGA NPs can enhance the cellular uptake of drug *via* endocytosis and may deliver drug to the target tissue/cell much more specifically *via* receptor-medicated endocytosis [6].

Originally used as an anti-fungal agent, rapamycin has been shown to be a potent anti-proliferative and anti-inflammatory drug which inhibits the mTOR-S6 Kinase 1 (S6K1) pathway [9]. Rapamycin also inhibits cell proliferation and inflammatory responses after angioplasty which are contributors to IH [10]–[12]. Intraluminal rapamycin-eluting stents are effective in suppressing IH, but detrimental late thrombosis develops due to the fact that locally released rapamycin also inhibits endothelial cells [13]–[15]. Moreover, patients treated with rapamycin-eluting stents still develop IH although to a lesser degree than bare metal stents. The potential use of NPs for the perivascular delivery of rapamycin to treat IH has not been fully explored. Rapamycin-loaded PLGA NPs (hereafter denoted as rapamycin-loaded NPs or rapamycin-NPs) may be potentially an ideal tool to provide sustained drug release to inhibit this process. Although several studies using animal models indicate that periadventitial application of rapamycin is a promising approach, currently there is no established method to provide sustained drug delivery after surgical procedures. Both rapamycin and PLGA are FDA approved, so it is relatively easy to translate these methods to clinical applications [12], [14].

Using rapamycin as a model drug and PLGA NPs as the drug carrier, we have developed a drug delivery system that provides combined benefits of periadventitial local drug administration (convenient for application at the time of open surgery) and prolonged drug release resulting in improved efficacy. We found *in* *vitro,* that NPs were readily taken up by SMCs, allowing for more sustained release of rapamycin, and that rapamycin-loaded NPs produced a more sustained inhibition of S6 kinase than rapamycin alone. *In vivo,* in a rat carotid injury model, rapamycin-loaded NPs led to a more sustained inhibition of S6K1, SMC proliferation, IH and restenosis compared to rapamycin alone. Importantly, treatment with rapamycin-loaded NPs did not affect reendothelialization. Our studies thus suggest that periadventitial application of rapamycin-loaded NPs has a potential to develop into an improved therapeutic strategy for treating restenosis at the time of open vascular reconstructions.

## Materials and Methods

### Ethics Statement

The experiments involving animal use were carried out in strict accordance with the recommendations in the Guide for the Care and Use of Laboratory Animals of the National Institutes of Health. The protocol (Permit Number: M02273) was approved by the Institutional Animal Care and Use Committee (IACUC) of the University of Wisconsin-Madison. All surgery was performed under isoflurane anesthesia (through inhaling, flow rate 2 ml/min), and all efforts were made to minimize suffering. Animals were euthanized in a chamber gradually filled with CO_2_.

### Materials

Rapamycin was purchased from LC Laboratories (Woburn, MA). Chloroform (HPLC grade) was from Acros Organics (Fair Lawn, NJ). DMEM and fetal bovine serum (FBS) were from Invitrogen (Calsbad, CA). Poly(D,L-lactide-co-glycolide) (PLGA, 50∶50, MW 40 to 75 kDa), poly (vinyl alcohol) (PVA, 95%, hydrolyzed, average MW 95 kDa), dimethyl sulfoxide (DMSO), Tween 80, paraformaldehyde, and FITC were from Sigma-Aldrich (St. Louis, MO). SDS gels (10% acrylamide) were from Bio-Rad (Hercules, CA). The FITC loaded NPs were a product from Phosphorex (Hopkinton, MA). The diameter of FITC-NPs is 220±30 nm; the PLA:PGA ratio of NPs is 50∶50. Kolliphor P407 (Poloxamer 407, a poly(ethylene oxide)-poly(propylene oxide)-poly(ethylene oxide) triblock copolymer) was kindly provided by BASF Corporation (Tarrytown, NY) and was used to prepare pluronic gel. Other reagents were purchased from Thermo Fisher Scientific (Fitchburg, WI) unless otherwise stated. TEM grids were purchased from Electron Microscopy Science (Hatfield, PA). The HPLC system is a product of Hitach High Technologies American, Inc. (Dallas, TX).

Rabbit anti-Ki67 antibody was from Abcam (Cambridge, MA); Rat anti-CD31 was from R&D Systems (Minneapolis, MN), antibodies to mTOR, phospho-S6 kinase-1 and S6 kinase-1 were from Cell Signaling Technologies (Danvers, MA); Alexa-468 conjugated secondary antibody was from Invitrogen (Carlsbad, CA). Fluorescence and bright field images were acquired using a Nikon Ti-U Eclipse microscope equipped with the Nikon Elements software packages. Microscopic images were processed and analyzed using the Image J software (NIH).

### Preparation of Rapamycin-loaded NPs

Rapamycin-loaded NPs were prepared using a single emulsion (w/o) method as previously described [16], [17]. Briefly, 12 mg of rapamycin and 65 mg of PLGA were added into a 100 ml flask. Subsequently, 6.5 ml of chloroform was added to the flask and stirred at 500 rpm at 40°C for 5 h. Thereafter, 26 ml of PVA water solution (3%) was added to the rapamycin/PLGA/chloroform solution followed by sonication using a probe sonicator (UP 100H from Hielscher) at 65% amplitude for 15 min. The resulting solution was stirred vigorously at room temperature for 6 days to evaporate the chloroform. Rapamycin-loaded NPs were collected *via* centrifugation at 22,800×*g* for 20 min at 4°C, and then freeze-dried and stored at −80°C in a desiccator. These rapamycin-NPs were found to be stable at least within a year. Blank PLGA NPs were prepared using a similar procedure without rapamycin.

### Characterization of the PLGA NPs

The size distribution of the PLGA NPs dispersed in deionized water was measured using dynamic light scattering (DLS) (Malvern Zetasizer Nano-ZS90, 633 nm laser) at 25.0°C in triplicates. The morphology of the PLGA NPs was studied using transmission electron microscopy (TEM, Tecnai T12 G2) at 120 kV. The PLGA NPs were diluted with deionized water and then deposited on a copper grid coated with carbon. The NPs were negatively stained with 1% phosphotungstic acid solution and dried at room temperature.

Rapamycin loading level and its release rate from the rapamycin-NPs were measured by a high-performance liquid chromatography (HPLC) using ultraviolet (UV) detection at 278 nm. Rapamycin concentration in solution was quantified according to a standard curve established with known concentrations of rapamycin.

### Localization of PLGA Nanoparticles in Cultured Vascular SMCs and in Injured Rat Carotid Arteries

Vascular smooth muscle cells (SMCs) were isolated from the thoracoabdominal aorta of male Sprague-Dawley rats based on a protocol described previously [18]. Cells were seeded in 4-well chamber slides with a density of 1×10^4^ cells/well in DMEM containing 10% FBS and cultured at 37°C overnight with 5% CO_2_ supplied. Then the culture media were changed to DMEM containing 2% FBS with 10 µg/ml fluorescein isothiocyanate (FITC)-loaded nanoparticles (FITC-NPs; 2% FITC loaded). The cells were then fixed by 4% paraformaldehyde at the indicated time points and imaged using fluorescence microscopy. For *in* *vivo* experiments, 1 mg of FITC-NPs was applied to the outside of the balloon-injured rat carotid artery as we have previously reported [19]. Carotid arteries were retrieved at the indicated time points and embedded in optimal cutting temperature compound (OCT); 5 µm frozen sections were prepared and imaged using a fluorescence microscope (200×).

### In Vitro Rapamycin Release Study

To prepare 30% pluronic gel, 3 grams of Poloxamer 407 were dissolved in 10 ml PBS buffer by stirring overnight in the cold room. The *in* *vitro* rapamycin release profiles from either rapamycin-loaded NPs dispersed in pluronic gel (rapamycin-loaded NPs) or free rapamycin directly dispersed in pluronic gel (rapamycin) were determined in PBS (pH 7.4) containing 0.2% Tween 80 as described [20]. Three mg of rapamycin-loaded NPs or 300 µg of rapamycin in 15 µl DMSO/H_2_O (v/v = 9/1) were dispersed in 300 µl of 30% pluronic solution contained in a microfuge tube on ice. The tube was then transferred to a 37°C incubator. After pluronic gel solidified at 37°C, 1 ml of PBS was added. At the indicated time points, microfuge tubes were spun at 22,800×*g* for 5 min to separate the supernatant from the PLGA NPs/gel mixture (recovery rate 99.6%); the supernatant was collected and replaced with fresh PBS buffer. The supernatant was filtered (membrane pore size 200 nm) to remove any uncollected NPs. The rapamycin concentration in the supernatant was then analyzed by HPLC. The drug release tests were conducted four times.

### Western Blot

Rapamycin or rapamycin-loaded NPs (15 µg rapamycin for both) dispersed in 100 µl pluronic gel were placed in dialysis tubes with a molecular weight cut off (MWCO) of 10,000 Daltons (Thermo Fisher Scientific; Davenport, IL) that was capable of retaining NPs but not rapamycin. Dialysis tubes were then incubated with cultured smooth muscle cells. Cell culture media were replaced with fresh media every 24 h. Cells were retrieved at the indicated time points and lysed in RIPA buffer (50 mM Tris-HCl, 150 mM NaCl, 1% Nonidet P-40, 0.1% sodium dodecyl sulfate, and 10 µg/ml aprotinin). For *in* *vivo* experiments, carotid arteries were collected 14 days after surgery and treatment, and homogenized in RIPA buffer. Thirty micrograms of proteins from each sample were separated by SDS-PAGE on 10% gels and then transferred to nitrocellulose membranes. Protein expression was assessed by immunoblotting with rabbit anti-phospho-S6K1 or anti-S6K1 antibodies (Cell Signaling, Boston, MA). After incubation with horseradish peroxidase-conjugated secondary antibodies, specific proteins bands on the membranes were visualized by using enhanced chemiluminescence reagents (Pierce, Davenport, IL).

### Cell Viability and Proliferation Assay

Cell proliferation was determined by modified 3-[4,5-dimethyl-thiazol-2-yl]-2,5-diphenyltetrazolium bromide (MTT) assay (Thermo Fisher Scientific; Davenport, IL). Rapamycin or rapamycin-loaded NPs (both 15 µg rapamycin) was mixed with 100 µl pluronic gel on ice, and then transferred into a microdialysis tube with a molecular weight cut off of 10,000 Dalton (Thermo Fisher Scientific; Davenport, IL). The dialysis media (1.5 ml) was collected (and stored) and replaced with fresh PBS buffer every day. Prior to rapamycin treatment, rat vascular SMCs were plated at 30–40% confluence on a 96-well plate and incubated overnight with 100 µl DMEM containing 10% FBS. Then 30 µl of the dialysis media collected at each time point was added to SMCs and cultured for 96 h. MTT solution (10 µl; 12 mM) in phenol red-free culture medium was added to each well and incubated at 37°C for 4 h followed by addition of 100 µl of the SDS-HCL solution. After incubation of the plate at 37°C for 4 h, absorbance was measured at 570 nm.

### Rat Carotid Artery Balloon Injury and in vivo Drug Delivery

Male Sprague-Dawley rats (∼350 g) underwent carotid artery balloon injury. Briefly, after induction of anesthesia with isofluorane, a longitudinal incision was made in the neck. A 2-F balloon catheter (Edwards Lifesciences, Irvine, CA) was inserted through the left external carotid artery and inflated to a pressure of 2 atm to simulate the angioplasty procedure. Blood flow was re-established after injury. Rapamycin or rapamycin-NPs (100 µg rapamycin per 100 g body weight) was dissolved in 300 µl of 30% pluronic gel which remained as liquid on ice. The pluronic gel solution was then applied around the outside of the injured segment of carotid artery [19]. The gel solidified immediately after exposure to body temperature.

### Tissue Processing

Animals were sacrificed and perfused with 4% paraformaldehyde at the pressure of 100 mmHg on day 14 or day 28 after surgery; then the carotid arteries were retrieved and processed for embedding and sectioning. Serial cross-sections were made at 50 µm intervals and used for histological analysis and immunostaining.

### Immunohistochemistry (IHC)

Paraffin-embedded artery sections were immunostained with rabbit anti-Ki67 antibody (Cambridge, MA) and detected using goat anti-rabbit HRP conjugate IgG, developed in 3,3′ diaminobenzidine (DAB) solution, and followed by a counterstain of hematoxylin.

Immunofluorescent staining was performed on paraffin-embedded sections with rat anti- CD31antibpody (R&D Systems, MN; 1∶400), signals were detected using donkey anti-rat Alexa Fluor 546 antibody (Invitrogen; Carlsbad, CA). DAPI was used to identify nuclei. Antibody controls included species-matched normal rabbit IgG antibodies.

### Quantification of IHC Results

Five stained tissue sections from each animal were used. On each section images were taken from six different fields (magnification 200×). The Ki67 positive cells were manually counted. The number of Ki67 positive cells in each 200× image was defined as Ki67 positive (cells) per high power field (HPF). The data were pooled to generate the mean and standard deviation for each animal. The means from each of 5 animals were averaged, and the standard error of the mean (SEM) was calculated for each group.

For quantification of reendothelialization, previously published methods were used with minor modifications [21], [22]. Briefly, the luminal perimeter and the percentage of this perimeter that stained for CD31 on serial sections (n = 5) were measured using NIH Image J. The percentage of reendothelialization was then scored from 1 to 5 (1: <20%; 2∶20 to 40%; 3∶40 to 60%; 4∶60 to 80%; 5∶80%–100%) and the scores were averaged.

### Morphometric Analysis

Morphometric study was performed using H&E-stained paraffin sections of the carotid arteries. The areas enclosed by the external elastic lamina (EEL), the internal elastic lamina (IEL), and the luminal area were measured using the NIH Image J software as previously described [23]. Intimal area (IEL area minus luminal area) and medial area (EEL area minus IEL area) and their ratio (I/M ratio) were then calculated. Five sections per animal were used and a mean ± SEM was derived from at least three independent experiments. Data were analyzed by one-way analysis of variance (ANOVA). If significant, the ANOVA was followed by Turkeys multiple comparison test. P values less than 0.05 are considered statistically significant.

## Results

### Preparation and Characterization of Rapamycin-loaded NPs

**Figure 1:**
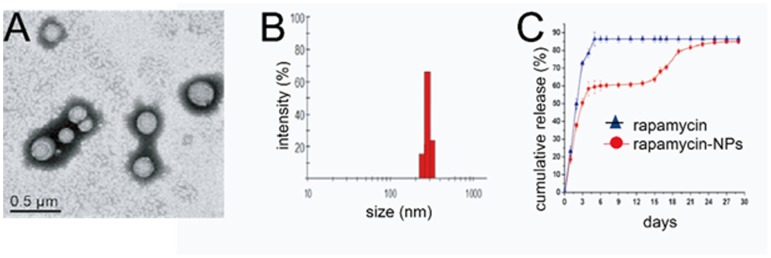
Characterization of rapamycin-loaded PLGA NPs (rapamycin-NPs) *in* *vitro*. (A). Transmission electron microscopy (TEM) image of rapamycin-NPs. (B). Size distribution of the rapamycin-NPs measured by dynamic light scattering analysis (DLS). (C). *In vitro* cumulative rapamycin release profiles from rapamycin-NPs (•, red) or free rapamycin (▴, blue), both encapsulated in pluronic gel immersed in PBS buffer. Data are presented as mean as mean ). (C). ing analysis (DLS). (C.

Rapamycin was encapsulated in PLGA NPs *via* a single emulsion method. PVA was coated on the surface of PLGA NPs to enhance their solubility/dispensability in aqueous solutions. The rapamycin encapsulation efficiency and loading level in the PLGA NPs were of 69.1% and 11.6%, respectively. Figure 1A shows a representative transmission electron microscopy (TEM) image of the rapamycin-loaded NPs. The average diameter of the NPs was around 250 nm. The size distribution of the rapamycin-loaded NPs measured by DLS (Figure 1B) ranged from 220 to 350 nm with an average diameter around 265 nm, which was in agreement with the TEM analysis.

To evaluate the comparative release of rapamycin or rapamycin-loaded NPs *in* *vitro,* both were dispersed in pluronic gel to mimic our *in* *vivo* model. The amount of released rapamycin was determined by HPLC. For rapamycin dispersed directly in pluronic gel, the amount of drug released after 2, 4, and 5 days was 50.0%, 78.4% and 86.4%, respectively. In contrast, sustained drug release was observed for 28 days from the rapamycin-loaded NPs dispersed in pluronic gel (Figure 1C). In both studies, pluronic gel dissolved after 3–4 days. The release of rapamycin from the rapamycin-NPs-Gel system exhibited a well-defined tri-phasic profile [20]. The initial burst of release of rapamycin during the first 4 days is likely attributed to the release of rapamycin near the surface of the PLGA NPs (Figure 1C). During the second phase of rapamycin release (5 to 15 days), rapamycin was released slowly from the PLGA NPs *via* a diffusion-controlled process [24], [25]. During the third phase (16 to 28 days), there was relatively rapid release of rapamycin again (16 to 21 days) likely attributable to the degradation and erosion of PLGA NPs [6], [26], [27], and then there was very minimal release of rapamycin after 21 days. These results demonstrate that rapamycin-loaded NPs dispersed in pluronic gel provide drug release in a more sustainable manner than rapamycin directly contained in a pluronic gel.

### Uptake of NPs by SMCs *in* *vitro* and After Periadventitial Application Around Injured Rat Carotid Arteries

**Figure 2:**
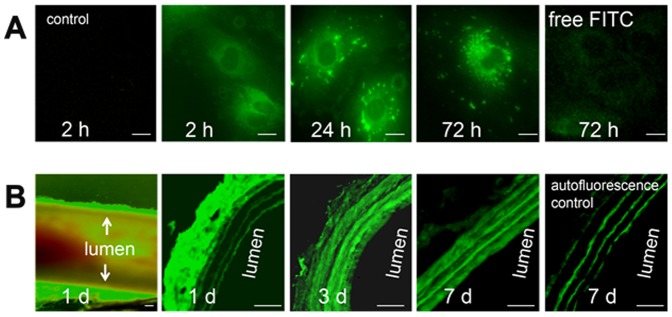
Accumulation of FITC-loaded NPs in cultured smooth muscle cells and in the arterial wall of the injured rat carotid artery after periadventitial application. (A). Representative fluorescence microscopic images demonstrate timein the arterial wall of the injured rat carotid arteµg FITC/ml) by cultured rat vascular smooth muscle cells (SMCs) (n = 3). Scale bar represents 10 µm. (B). FITC-NPs were applied around the rat carotid artery immediately after injury (see methods). Representative fluorescence microscopic images of carotid arteries demonstrate the in vivo distribution of FITC-NPs *(n = 3)* (1 mg FITC-NPs in 300 µl pluronic gel/artery). The first panel of B is a low-magnification longitudinal image of the artery showing perivascular application of NPs. Panels 2–4 are images of cross sections. The last panel shows the auto-fluorescence background of laminas. Scale bar represents 120 µm.

To evaluate the cellular absorption and distribution of NPs *in* *vitro,* FITC-loaded PLGA NPs (FITC-NPs) were applied to cultured vascular SMCs as described in Methods. As is evident in Figure 2A, FITC-NPs were readily taken up by SMCs as early as 2 hours and NPs accumulated primarily in the cytoplasm. Punctate collections of nanoparticles were observed by 24 hours. We then investigated whether PLGA NPs could be readily dispersed into the arterial wall. FITC-NPs in pluronic gel were applied to the adventitia of rat carotid arteries immediately after balloon injury. We found at 24 h, FITC-NPs were localized around and within the adventitia of the injured carotid arteries (Figure 2B). At 72 h, after dissolution of the pluronic gel, FITC-NPs had migrated into the arterial wall as well as into the loose connective tissues that surround the artery (Figure 2B). Only a small portion of FITC-NPs was located in the arterial wall compared to the total applied amount, probably because of fast dissolution of pluronic gel, emphasizing the need to develop long-lasting gels in the future for *in* *vivo* applications.

### Rapamycin-loaded NPs Produce Sustained Drug Release *in* *vitro* and *in* *vivo* in Balloon-injured Rat Carotid Arteries

**Figure 3:**
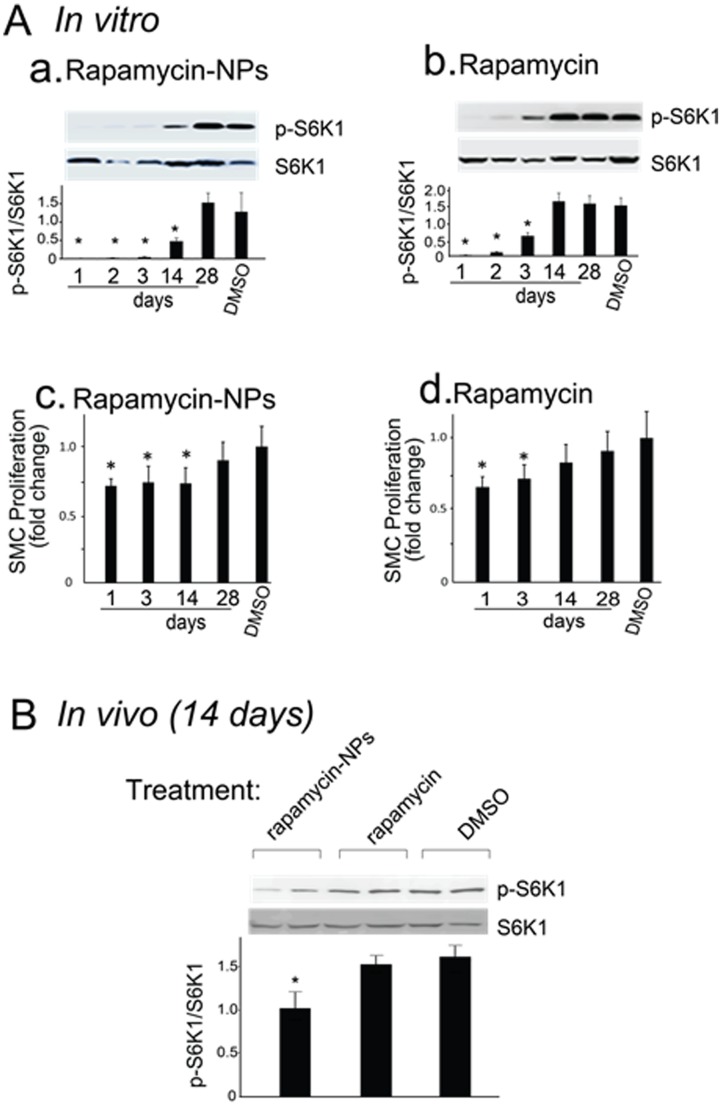
Prolonged inhibitory effects of rapamycin-loaded NPs on S6K1 phosphorylation *in* *vitro* and *in* *vivo*. (A). *In vitro experiments*. Treatment of SMCs with Rapamycin or rapamycin-NPs (15 µg rapamycin for both) is described in detail in Materials and Methods. Panels a and b show the effect of rapamycin-NPs and rapamycin on p-S6K, respectively. Proteins were extracted from SMCs at the indicated time points, and phosphorylated S6K1 (p-S6K1) and S6K1 were measured by Western blot analysis. Panels c and d show the effect of rapamycin-NPs and rapamycin on SMC proliferation (measured by MTT assay), respectively. Quantified data are presented as mean MC proliferation (measured by MTT (* P<0.05). (B). *In vivo experiments*. Following balloon angioplasty in rat carotid arteries, rapamycin or rapamycin-NPs (300 µg rapamycin for both) were dispersed in 300 µl pluronic gel and applied periadventitially to injured carotid arteries, as described in Methods. Carotid arteries were retrieved 14 days after surgery. Proteins extracted from carotid arteries were subjected to Western blot analysis for phosphorylated S6K1 (p-S6K1) and S6K1. Quantified data are presented as mean njured carotid arteries, as descri (* P<0.05).

After confirming uptake of NPs by SMCs *in* *vitro* and distribution into the arterial wall *in* *vivo*, we next examined the functional effect of drug release from rapamycin-loaded NPs on SMCs *in* *vitro* and on cells of the arterial wall. Previous studies have shown that rapamycin halts cell cycle progression by specifically targeting the mTOR pathway and inhibiting the phosphorylation of downstream S6K1 [9], [28]. We first compared the inhibitory effect of rapamycin-loaded NPs with that of free rapamycin on S6K1 phosphorylation in cultured SMCs. Rapamycin or rapamycin-loaded NPs dispersed in pluronic gel were placed in a dialysis tube, capable of retaining NPs but allowing the release of rapamycin into the culture dish. Cells were seeded with a low density to allow their long term viability. Cell culture media were replaced with fresh media every 24 h. SMCs were then collected at the specified time points, and S6K1 phosphorylation was evaluated by Western blotting. Whereas free rapamycin inhibited S6K1 phosphorylation for only 3 days following treatment, rapamycin-loaded NPs significantly suppressed S6K1 phosphorylation for up to 14 days (Figure 3A, panels a and b). A similar pattern was observed regarding the effect of rapamycin-loaded NPs and rapamycin on SMC proliferation (Figure 3A, panels c and d). These results suggest that rapamycin-loaded NPs facilitated prolonged drug release which produced a sustained inhibitory effect on SMC function as evidenced by S6K phosphorylation.

Since the *in* *vitro* data indicated that on day 14 rapamycin-loaded NPs but not free rapamycin suppressed S6K1 phosphorylation (Figure 3A), we then assessed using that same time point whether perivascular application of rapamycin-loaded NPs provided prolonged drug release into the arterial wall compared to rapamycin alone (Figure 3B). We again used S6K1 phosphorylation as a surrogate for measuring rapamycin’s functional effect. We placed rapamycin-loaded NPs or rapamycin alone in pluronic gel, which was then applied to the outside of balloon-injured rat carotid arteries. Arteries were retrieved 14 days after balloon injury and drug application. Proteins were then extracted and Western blotting was performed to examine S6K1 phosphorylation. Consistent with our *in* *vitro* findings, S6K1 phosphorylation on day 14 following treatment was reduced by ∼70% in the rapamycin-NPs-treated group, however no significant effect was observed in the group treated with rapamycin alone (Figure 3B). Together, our data demonstrate that rapamycin-loaded NPs versus free rapamycin, provide sustained inhibition of S6K1 phosphorylation in SMCs both *in* *vitro* and *in* *vivo*. These findings suggest that a drug delivery strategy employing NPs can provide prolonged drug release with more sustained functional effects.

### Periadventitial Administration of Rapamycin-loaded NPs Produces Sustained Inhibition of Intimal Hyperplasia (IH)

**Figure 4:**
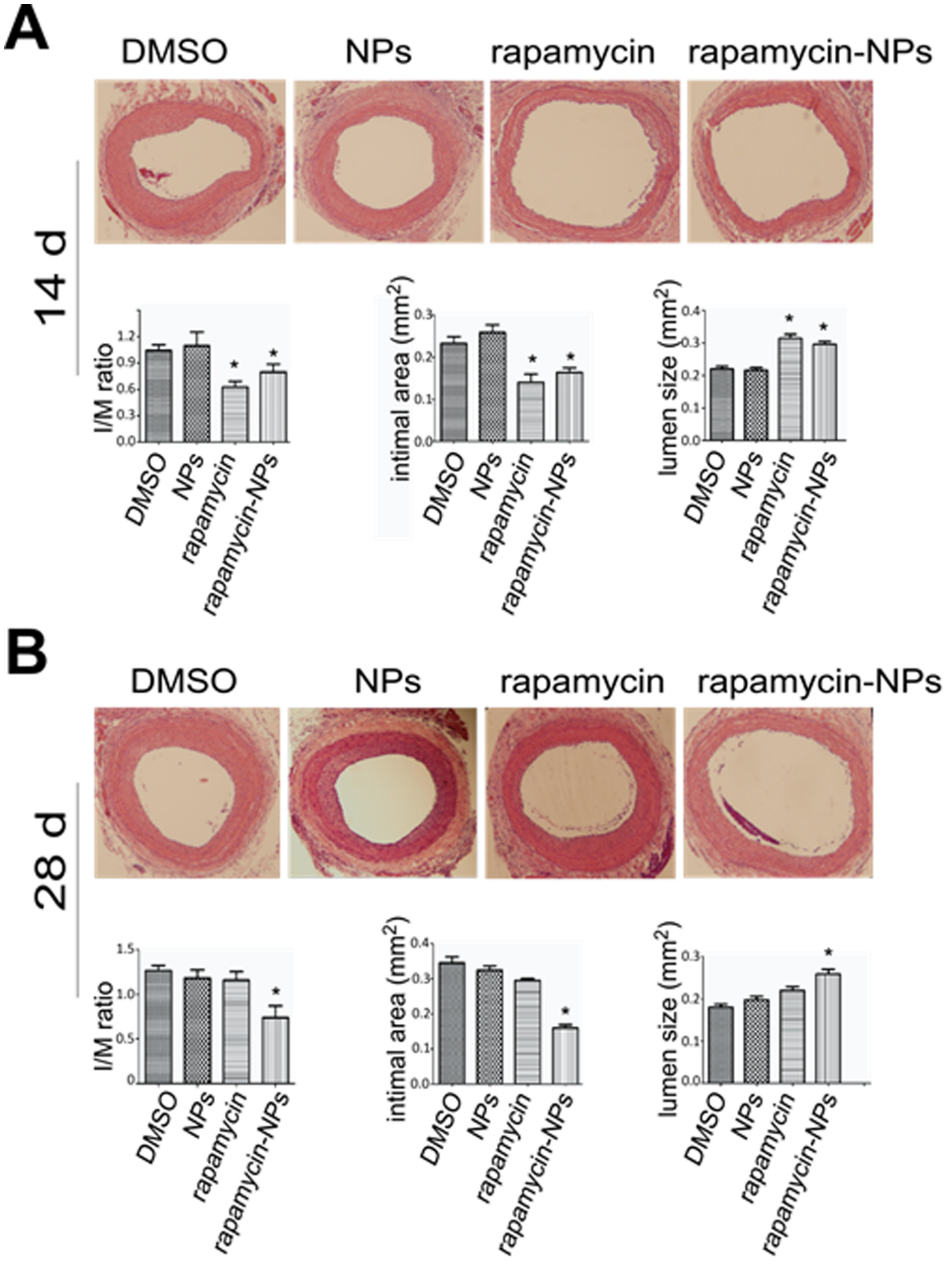
Sustained inhibitory effects of rapamycin-loaded NPs on intimal hyperplasia in balloon-injured rat carotid arteries. Balloon injury of rat carotid arteries was performed and rapamycin or rapamycin loaded NPs (300 µg rapamycin for both) dispersed in 300 µl pluronic gel was applied periadventitially immediately after vascular injury, as described in Methods. Solvent (DMSO) and NPs alone dispersed in pluronic gel were used as controls. Carotid arteries were retrieved 14 (A) or 28 days (B) after surgery. Sections were then prepared and H&E stained. Top panel shows representative microscopic images of carotid cross-sections from the indicated treatment groups. Bottom panel shows quantification of lumen size, intimal area, and intimal to media ratio (I/M). Data are presented as mean ± SEM from 5 animals in each group (*P<0.05 compared to DMSO control).

Encouraged by the observed sustained functional effect of rapamycin-loaded NPs compared to free rapamycin, we evaluated the comparative ability of both approaches to inhibit intimal hyperplasia in a rat carotid artery injury model. Rapamycin-loaded NPs or free rapamycin were placed in pluronic gel and applied around the carotid artery immediately following balloon injury. For controls, we applied the solvent for rapamycin (DMSO) or unloaded NPs in pluronic gel. Arteries were retrieved 14 days or 28 days following treatment. As shown in Figure 4A at 14 days compared to both controls, intimal hyperplasia (measured by the I/M ratio) was markedly reduced and the arterial lumen was significantly greater in arteries treated with either rapamycin alone or rapamycin-loaded NPs. Thus, at 14 days both treatments were equally effective. Twenty-eight days after treatment there was sustained inhibition of IH and maintenance of lumen size in animals treated with rapamycin-loaded NPs. However, in animals treated with free rapamycin in pluronic gel, IH returned to a level similar to untreated controls with a corresponding diminution in lumen diameter (Figure 4B). These results suggest that sustained drug release from rapamycin-loaded NPs prolonged the inhibitory effect of rapamycin on IH leading to a durably patent vessel following arterial injury.

### Rapamycin-loaded NPs Provide Prolonged Inhibition of Cellular Proliferation in Media and Subintima of Injured Rat Carotid Arteries

**Figure 5:**
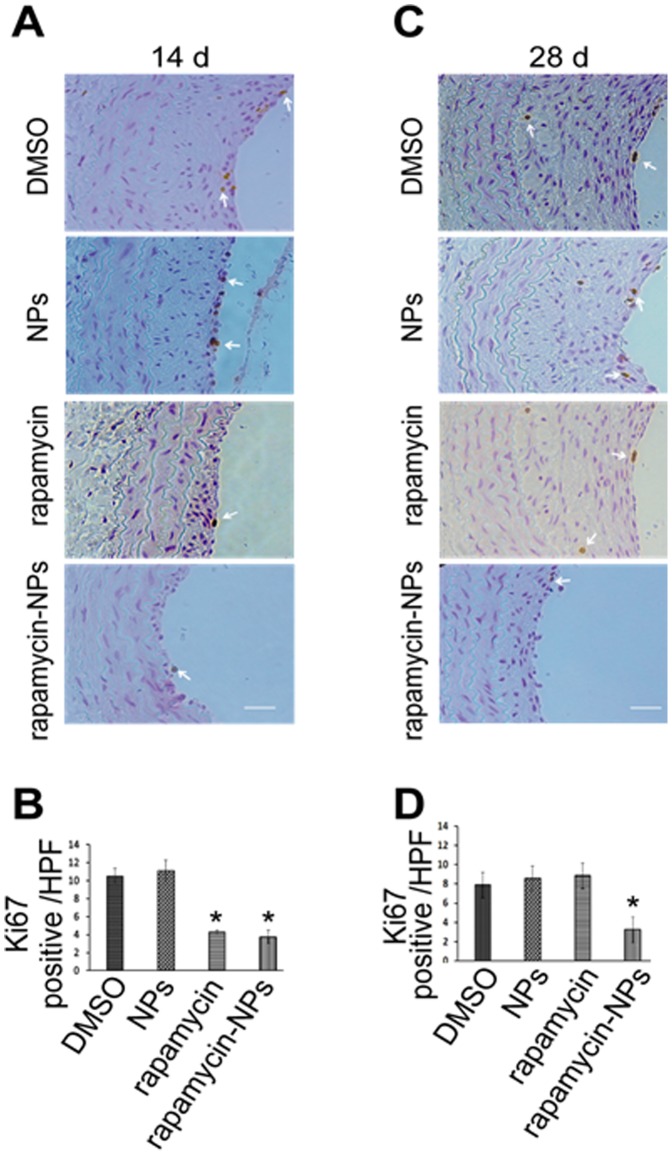
Sustained inhibitory effect of rapamycin-NPs on cell proliferation in balloon-injured rat carotid arteries. Rat carotid cross-sections were obtained from the same experiments as in Figure 4. Sections were immunostained for Ki67 as described in Methods. Representative microscopic images of Ki67 staining on arteries retrieved 14 days (A) and 28 days (C) after surgery. Arrows point to Ki67 positive cells. Quantification of Ki67 positive cell number per high power field (HPF) on sections retrieved 14 (B) and 28 days (D) after surgery (magnification is 200X). Each bar represents a mean ±SEM of 5 animals (* P<0.05 compared to DMSO control).

Previous studies have indicated that rapamycin impedes IH at least in part by effectively inhibiting SMC proliferation [29]. In these studies we investigate the mechanism underlying the attenuation of IH by rapamycin-NPs. Using histological sections from the animals treated in the foregoing experiments, immunostaining for Ki67 was performed to evaluate cell proliferation. Our data reveal that the proliferation or Ki67 index was substantially decreased (by ∼50%) in both the free rapamycin and rapamycin-NPs treated groups compared to controls at 14 days following treatment (Figure 5, A and B). However 28 days after treatment, whereas the Ki67 index remained suppressed in the rapamycin-NPs treated group, the Ki67 index in the free rapamycin-treated group returned to the level of control (Figure 5, C and D). These results demonstrate that compared to rapamycin alone, the use of NPs to locally deliver rapamycin produced a prolonged inhibitory effect on cell proliferation in balloon-injured arteries likely accounting for the sustained inhibition of IH (Figure 4).

### Rapamycin-loaded NPs Have no Effect on Reendothelialization of Injured Rat Carotid Arteries

**Figure 6:**
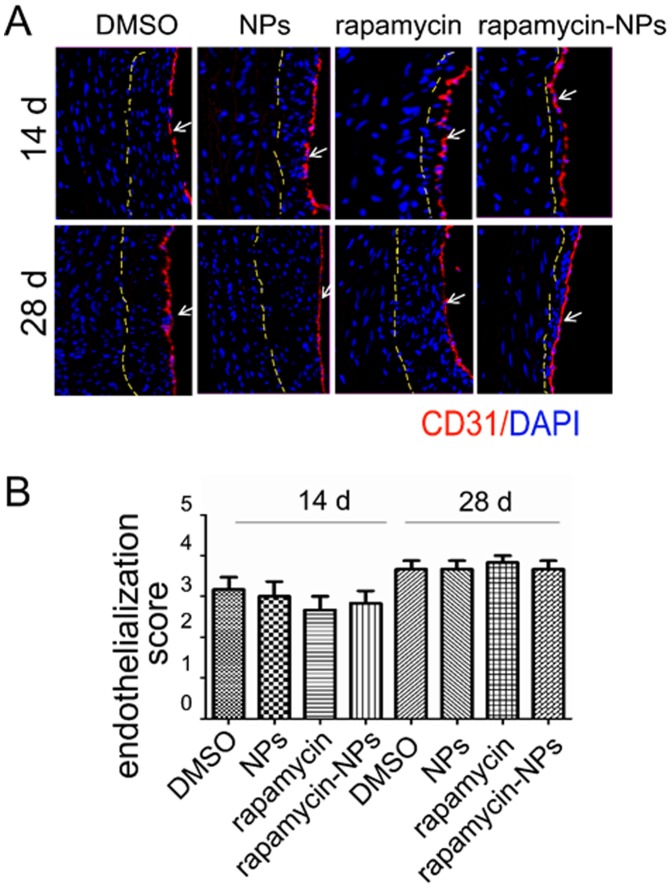
Periadventitial application of rapamycin-loaded NPs does not affect carotid artery reendothelialization after angioplasty. Rat carotid cross-sections were obtained from the same experiments as in Figure 4. (A) Representative fluorescence microscopic images of CD-31staining (red, marked by arrows) of arteries retrieved 14 or 28 days after surgery. Blue dots are DAPI-stained nuclei. Dashed lines define internal elastic lamina (IEL). (B) Quantification of reendothelialization (CD-31 positive versus total perimeter) on sections retrieved 14 or 28 days after surgery. Each bar represents a mean ±SEM of 5 animals.

It has been well demonstrated that rapamycin-eluting stents inhibit endothelial cell proliferation and thus delay reendothelialization, producing the adverse side effect of acute vascular thrombosis [30]. To evaluate the potential for periadventially applied rapamycin-loaded NPs to inhibit reendothelialization, we performed immunostaining for CD31 (a marker for endothelial cells) on carotid sections derived from animals treated with rapamycin-NPs, free rapamycin or controls. We found equivalent rates of reendothelialization in animals treated with rapamycin-NPs compared to controls (Figure 6). Moreover, the rate of reendothelialization was also not diminished in arteries treated with free rapamycin in pluronic gel. These findings were verified at both 14 and 28 days after injury. Thus rapamycin when applied to the arterial adventitia does not affect the regrowth rate of the endothelial layer.

Finally, we evaluated whether rapamycin-loaded NPs provide systemic toxicity to treated animals. We found that periadventitial application of either rapamycin alone or rapamycin-loaded NPs had no effect on weight gain (Supplemental Figure 1) or blood cell counts (Supplemental Tables 1 and 2) during the course of these experiments. Thus, local application of rapamycin externally to the artery wall, is a safe method for sustained drug release without systemic effects.

## Discussion

Through periadventitial application of rapamycin-loaded NPs, we have achieved prolonged attenuation of intimal hyperplasia (IH) for at least 4 weeks while avoiding impairment of endothelialization. Our *in* *vitro* and *in* *vivo* experiments together indicate that NPs facilitate prolonged release of rapamycin. Persuasive evidence can be summarized as follows. Rapamycin-loaded NPs produce sustained drug release for up to 28 days compared to 3–5 days with free rapamycin. Rapamycin-loaded NPs produce prolonged inhibition of S6K1 phosphorylation (14 days) in cultured SMCs compared to rapamycin alone (3 days). It is well established that rapamycin inhibits mTOR leading to decreased downstream S6K1 phosphorylation, which is required for cell cycle progression and cell proliferation [10], [31]. Thus, phospho-S6K1 is widely used as a valid functional marker for rapamycin bioavailability. Using phospho-S6K1 inhibition as an indicator, we found that rapamycin-loaded NPs applied periadventitially promoted prolonged release of rapamycin into the vessel wall compared to rapamycin alone. Finally, rapamycin-loaded NPs applied around the injured artery greatly outperformed rapamycin alone in durable inhibition of SMC proliferation and IH. These lines of evidence clearly show that NPs improve the durability of drug release and prolong the effect of rapamycin in impeding IH.

Currently the only clinically applied methods for treating IH are stents or balloons that release anti-proliferative drugs such as rapamycin or paclitaxel [1], [32]. Drug-eluting stents have been successful in reducing the incidence of IH in patients treated with coronary artery angioplasty although there are limitations including the need for chronic platelet inhibition, the potential for stent thrombosis related to delayed endothelialization, as well as cost. Despite advances made over the past two decades in percutaneous angioplasty, thousands of patients each year are still treated with traditional open surgery. These procedures include lower extremity bypass both vein and prosthetic, coronary artery bypass and carotid endarterectomy (total up to 270 thousand cases per year) and vascular grafts placed for dialysis (∼50 thousand cases per year) [33]. Unfortunately drug-eluting stents or balloons used following percutaneous angioplasty are not applicable for the patients undergoing open surgical procedures. There are currently no available measures to prevent the development of IH in these patients where the incidence ranges from 20–80%. Thus, there is a tremendous unmet clinical need for a clinically applicable technique to prevent IH in patients undergoing open vascular reconstructive surgery.

Since angioplasty is performed remotely, intraluminal drugs applied to the site of angioplasty must also be delivered remotely. Thus the challenges of drug delivery following a percutaneous intervention are substantial. Alternatively, drugs that inhibit IH can be directly applied to bypass grafts, anastomoses, prosthetic grafts, or endarterectomized arteries. The task of drug delivery following open surgery is conceptually less challenging since drugs can be directly applied to the arterial wall. In addition to the accessibility of the vessel at the time of surgery there are other distinct advantages of applying drugs to the arterial adventitia. Inhibitors of SMC proliferation also inhibit endothelial cell proliferation. Interestingly, rapamycin inhibits endothelial cells to a much greater extent than SMCs. Thus, rapamycin when applied intraluminally *via* a stent markedly inhibits endothelial cell proliferation leading to loss of the endothelial lining, the potential for acute vessel thrombosis and the need for prolonged platelet inhibition [1], [34]. Periadventitial application of rapamycin has the potential of creating a gradient so that the greatest concentrations of rapamycin influence the adventitia followed by the media and the subintima with intimal endothelial cells exposed to the lowest concentration of drugs. To this end we studied reendothelialization in our model and found that when rapamycin was applied to the periadventitial tissue there was rapid endothelial regeneration without rapamycin’s usual inhibitory effect. An additional theoretical advantage of periadventitial application of drugs that inhibit cellular proliferation is the ability of these drugs to influence cells in the adventitia. Myofibroblasts in the adventitia significantly contribute to neointimal hyperplasia. There is extensive data demonstrating that adventitial myofibroblasts migrate to the subintima and contribute to the formation of plaque [35], [36]. Thus inhibition of adventitial myofibroblast proliferation may enhance the ability of drugs such as rapamycin to protect against IH. With an effective method of drug delivery, the potential to reduce morbidity and mortality associated with recurrent disease, graft failure and vascular occlusion is substantial.

There is mounting evidence that prolonged drug delivery may be advantageous. There are various stimuli after vascular reconstruction that lead to the development of IH. Inclusive is vessel wall damage that accompanies manipulation and suture of a graft to the vessel wall. Veins used for bypass during harvest are exposed to trauma, desiccation, over-distention and *ex vivo* preservation. Endarterectomy produces direct trauma to the arterial media. Although these events are transient, the degree of injury can be profound leading to a protracted course of healing. A short burst of drug may be inadequate to completely block the maladaptive healing response that leads to IH. Moreover, some vascular reconstruction procedures are associated with an ongoing stimulus for IH. Following the creation of a bypass, “low pressure” veins are subject to arterial pressure, which provides a persistent (for the life of the bypass) stimulus for arterial remodeling that can lead to IH. Altered flow dynamics at the site of an anastomosis are persistent and likewise provide an ongoing hyperplastic stimulus. Grafts for bypass eventually reach a steady state of adaptive healing; however, this is likely not to be achieved for at least 3 to 6 months following the initial reconstruction. Thus prolonged delivery of drug over a several months period of time would seem advantageous.

Many of the pharmacological properties of conventional or “free” drugs can be improved through the use of NP drug delivery systems. First, NP drug delivery systems can significantly enhance the solubility of hydrophobic drugs in aqueous solutions. Encapsulating rapamycin in the PLGA NPs can significantly enhance its concentration in an aqueous solution [6]. Second, NPs provide controlled and sustained drug release profiles. Our *in* *vitro* data revealed that free rapamycin in pluronic gel was released over a period of 3 to 5 days whereas rapamycin in PLGA NPs was released over 3–4 weeks. Moreover, S6K1 phosphorylation and cell proliferation were effectively suppressed for at least 28 days in animals treated with rapamycin-loaded nanoparticles. It is likely that sustained release of rapamycin was a major factor that led to more durable inhibition of IH in our model. Another mechanism through which NPs can promote drug delivery is by enhancing the accumulation and absorption of drug by target cells or tissues. NPs can release rapamycin into the extracellular space and then free rapamycin can diffuse into cells of the arterial wall. NPs can also be taken up readily by cells *via* endocytosis, due to their relatively small size (∼200 nm *vs.* SMCs being ∼20 micrometers in size) [6]. In our *in* *vitro* system we observed that NPs were internalized by SMCs *via* endocytosis within two hours. The relative cellular uptake of free drug versus NPs varies and depends on the cell type, the nature of the drug, as well as the chemical, physical, and surface properties of the NPs (*e.g.,* specific ligand-receptor interaction) [37]. In our system it is clear that rapamycin is able to enter the cell *via* both mechanisms. It is worth noting that once NPs are exposed to cell culture medium, proteins/peptides present in cell culture may coat the surface of the NPs, which later serve as a targeting ligand promoting cellular uptake of NPs [37]. Lastly, NPs prevent drug from premature enzymatic degradation *in* *vivo.* The *in* *vivo* half-life of free rapamycin is much shorter than *in* *vitro*. However, rapamycin encapsulated in NPs is protected from any enzymatic degradation until it is released from the NPs.

While many strategies involving NPs have been designed for the luminal treatment of restenosis, intraluminal approaches have the potential to cause undesirable inhibition of the inner endothelial protective lining [1]. A number of approaches for perivascular drug delivery of rapamycin have been employed but with varying success. These approaches include rapamycin-loaded microbeads, PLGA membranes, synthetic meshes, and non-constrictive cuffs [7], [38], [39]. Recently, periadventitial application of rapamycin-eluting microbeads (200 µm) was evaluated in a pig vein graft model. Low concentrations of rapamycin only partially inhibited IH whereas higher concentrations produced anastamotic disruption related to absent healing [40]. NPs have the advantage over these other approaches in penetrating the arterial wall allowing more direct cellular delivery.

Although our study has clearly demonstrated that perivascular application of rapamycin-loaded PLGA NPs is effective in the inhibition of IH, further research is needed to optimize this drug delivery system. For example, we were not able to evaluate whether rapamycin-loaded NPs might affect IH beyond 4 weeks. A longer-term model of IH will be necessary to accomplish this evaluation. The current NP drug delivery system can also be improved through a number of manipulations. Pluronic gel was used to immobilize NPs in the periadventitial space. However, pluronic gel dissolves within 3–4 days after *in* *vivo* application. Using a long-lasting yet biodegradable temperature-responsive polymer gel to replace pluronic gel will further extend *in* *vivo* drug release from NPs. Moreover, NPs can be modified to meet specific needs for optimal drug release. Polymer chemistry and size of NPs can be altered to vary NP durability, and drug release profile. In addition, various ligands can be conjugated to the surface of the NPs to target specific cell populations overexpressing corresponding receptors in the hyperplastic vessel wall. Lastly, NPs are capable of encapsulating a mixture of drugs with complementary functions, which may further enhance the efficacy of drug-releasing NPs for treating restenosis. Further studies with new polymer gels, drug nanocarriers, animal models, and multi-drug administration regimens should be able to further improve the efficacy of drug delivery.

### Conclusion

Our study shows that periadventitial delivery of rapamycin-loaded NPs is a promising approach for the development of a safer, more efficacious drug delivery system to treat IH. Using rapamycin as a model drug we have demonstrated that NPs extend drug release *in* *vitro* and *in* *vivo*. When applied outside the arterial wall, rapamycin-loaded NPs compared to rapamycin alone substantially prolonged inhibition of IH and maintained lumen patency in balloon-injured rat carotid arteries. Thus local drug delivery with NPs provides a useful template approach for future development of safe and efficacious drug delivery methods to treat IH and restenosis, particularly for patients undergoing open vascular reconstruction.

## Supporting Information

Figure S1 **Periadventitial application of rapamycin-loaded NPs does not affect body weight.** Animal body weights were measured at the indicated time points after surgery. Data are presented as mean sents a mean ±SEM of 5 animals.CD-31s.
(TIF)

Table S1 **Periadventitial application of rapamycin-loaded nanoparticles does not affect blood cell counts (14 days after Surgery).** Hematological analysis from Sprague Dawley Rats 14 d after surgery. Results are expressed as mean ± SD, n = 5. RBC, red blood cells; WBC, white blood cells.
(TIF)

Table S2 **Periadventitial application of rapamycin-loaded nanoparticles does not affect blood cell counts (28 days after Surgery).** Hematological analysis from Sprague Dawley Rats 14 d after surgery. Results are expressed as mean ± SD, n = 5. RBC, red blood cells; WBC, white blood cells.
(TIF)
