# Supplementary figures and images for: Periadventitial Application of Rapamycin-Loaded Nanoparticles Produces Sustained Inhibition of Vascular Restenosis
Source: PLoS One. 2014 Feb 21;9(2):e89227. doi: 10.1371/journal.pone.0089227 (PMC3931710; doi:10.1371/journal.pone.0089227)

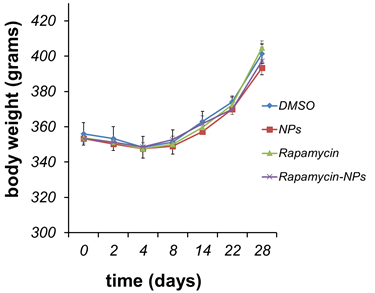

Supplement: Figure S1 — Periadventitial application of rapamycin-loaded NPs does not affect body weight. Animal body weights were measured at the indicated time points after surgery. Data are presented as mean sents a mean ±SEM of 5 animals.CD-31s. (TIF) [file pone.0089227.s001.tif]

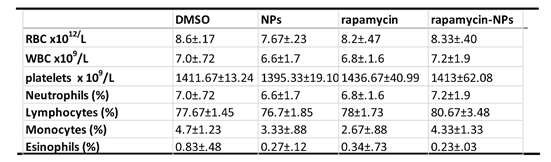

Supplement: Table S1 — Periadventitial application of rapamycin-loaded nanoparticles does not affect blood cell counts (14 days after Surgery). Hematological analysis from Sprague Dawley Rats 14 d after surgery. Results are expressed as mean ± SD, n = 5. RBC, red blood cells; WBC, white blood cells. (TIF) [file pone.0089227.s002.tif]

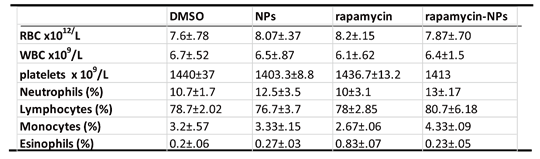

Supplement: Table S2 — Periadventitial application of rapamycin-loaded nanoparticles does not affect blood cell counts (28 days after Surgery). Hematological analysis from Sprague Dawley Rats 14 d after surgery. Results are expressed as mean ± SD, n = 5. RBC, red blood cells; WBC, white blood cells. (TIF) [file pone.0089227.s003.tif]
